# ^1^H, ^15^N, ^13^C resonance assignments for proteasome shuttle factor hHR23a

**DOI:** 10.21203/rs.3.rs-3256627/v1

**Published:** 2023-08-17

**Authors:** Xiang Chen, Kylie Walters

**Affiliations:** National Cancer Institute

**Keywords:** Ubiquitin-proteasome pathway, DNA repair protein, shuttle factor, Rad23, NMR

## Abstract

hHR23a (human homolog of Rad23 a) functions in nucleotide excision repair and proteasome-mediated protein degradation. It contains an N-terminal ubiquitin-like (UBL) domain, an xeroderma pigmentosum C (XPC)-binding domain, and a ubiquitin-associated (UBA) domain preceding and following the XPC-binding domain. Each of the four structural domains are connected by flexible linker regions. We report in this NMR study, the ^1^H, ^15^N and ^13^C resonance assignments for the backbone and sidechain atoms of the hHR23a full-length protein with BioMagResBank accession number 52059. Assignments are 97% and 87% for the backbone (^N^H, N, C’, Cα, and Hα) and sidechain atoms of the hHR23a structured regions. The secondary structural elements predicted from the NMR data fit well to the hHR23a NMR structure. The assignments described in this manuscript can be used to apply NMR for studies of hHR23a with its binding partners.

## Biological context

hHR23a (human homolog of Rad23 a) is one of two human orthologs of yeast radiation sensitivity abnormal 23 (Rad23) that binds to xeroderma pigmentosum C (XPC), forming a complex that functions in nucleotide excision repair of bulky lesions in DNA, such as induced by ultraviolet light exposure (Dantuma et al., 2009). It also acts as a shuttle factor in delivering ubiquitinated proteins to the proteasome (Chen et al., 2021; Osei-Amponsa and Walters, 2022; Wang et al., 2003) and in the nucleus, the two UBA domains of hHR23b have been shown to drive the formation of proteasome-containing biomolecular condensates (Yasuda et al., 2020). hHR23a comprises four well defined functional domains connected by flexible linker regions: an N-terminal ubiquitin-like (UBL) domain followed by an internal ubiquitin-associated (UBA) domain (UBA1), an XPC-binding domain, and a C-terminal UBA domain (UBA2) (Walters et al., 2003). As a ubiquitin-binding shuttle factor, hHR23a/Rad23 recognizes ubiquitinated substrates with its two UBA domains (Bertolaet et al., 2001; Wang et al., 2003) and the proteasome subunits Rpn1, Rpn10 and Rpn13 with its UBL domain (Elsasser et al., 2002; Husnjak et al., 2008; Rosenzweig et al., 2012; Gomez et al., 2011; Mueller and Feigon, 2003; Shi et al., 2016; Chen et al., 2016). The hHR23a UBL domain interacts dynamically with its two UBA domains and this interaction is broken when hHR23a binds to either proteasome component Rpn10 or ubiquitin (Walters et al., 2003; Wang et al., 2003). hHR23a can also bind another shuttle factor UBQLN2 and form heterodimer by UBL/UBA domain interactions (Kang et al., 2007b). The structure of hHR23a has been extensively studied (Dieckmann et al., 1998; Withers-Ward et al., 2000; Mueller and Feigon, 2002; 2003; Walters et al., 2003; Wang et al., 2003; Kamionka and Feigon, 2004; Kang et al., 2007a; Jiang et al., 2017; Byeon et al., 2021), but NMR assignments of the full protein are not available, with availability of only the individual UBA2 domain (BMRB 4757)(Withers-Ward et al., 2000), UBA1 domain (PDB 5XBO)(Jiang et al., 2017), and a construct that includes the XPC-binding and UBA2 domains (BMRB 27978)(Byeon et al., 2020). Here, we report the chemical shift assignments for full-length hHR23a, which is related to our previous reported full-length hHR23a NMR structure (Walters et al., 2003). These assignments may serve as a foundation for NMR studies of hHR23a interactions with proteasome subunits Rpn1, Rpn10, and Rpn13 or other ubiquitin receptors, such as Ddi1 or UBQLN2.

## Methods and experiments

### Expression and purification of hHR23a

Full-length hHR23a was subcloned between BamHI and NotI restriction sites of the pGEX-6P-1 vector (Cytiva 27-1542-01) in frame with an N-terminal glutathione S-transferase (GST) and a PreScission protease cleavage site. The plasmid was transformed into *E. coli* strain BL21(DE3) (Thermo Fisher Scientific C600003) with selection by ampicillin. The transformed colonies were grown in 10 mL of Luria-Bertani Broth (LB) medium (ampicillin 100 μg/ml) overnight at 37°C with shaking and centrifuged for 10 minutes at 2,000 g. The bacterial pellets were then gently resuspended and diluted at 1:100 ratio with 1 L of M9 minimal media supplemented with 1 g/L ^15^N ammonium chloride (Sigma-Aldrich) and 3 g/L ^13^C glucose (Sigma-Aldrich) as the sole nitrogen and carbon sources, or 1 g/L unlabeled ammonium chloride and 3 g/L ^13^C glucose, or 1 g/L ^15^N ammonium chloride and 3 g/L ^13^C glucose in 50% ^2^H_2_O (Sigma-Aldrich) / 50% ^1^H_2_O, as described in (Chen and Walters, 2012). Cells were grown at 37°C with shaking until they reached an OD_600_ of 0.5–0.6 at which point isopropyl β-D-1-thiogalactopyranoside (UBPBio) was added to a final concentration of 0.4 mM to induce protein expression at 17°C overnight. The bacteria were harvested by spinning down for 30 minutes at 5,000 g and 4°C in a Beckman Coulter J6-M1 centrifuge with a JS-4.2 rotor. The harvested cell pellets were frozen in liquid nitrogen and stored at −80°C until purification.

Cell pellets were resuspended in lysis buffer (20 mM NaPO_4_ at pH 6.5, 300 mM NaCl, 2 mM DTT) supplemented with protease inhibitor cocktail tablets (Roche Diagnostics 11836153001). Resuspended cells were sonicated and centrifuged for 30 min at 27,000 g and 4°C. The supernatant was incubated with pre-washed glutathione-Sepharose beads (Cytiva 17075605) for 3 hours at 4°C with agitation. The beads were washed extensively in lysis buffer and incubated overnight with PreScission protease (Cytiva 27084301) at 4°C with agitation to release hHR23a protein from the GST tag. hHR23a proteins were eluted from the beads in lysis buffer and further purification was achieved by size exclusion chromatography on an FPLC ÄKTA pure system (Cytiva) equipped with a HiLoad 16/600 Superdex 200 prep grade column (Cytiva) in FPLC buffer (20 mM NaPO_4_ at pH 6.5, 100 mM NaCl, 2 mM DTT). hHR23a proteins were concentrated by Amicon Ultra-15 filters with a 3 kDa cutoff (EMD Millipore UFC900324) to ~ 0.5 mM.

### NMR experiments

Three NMR samples were prepared, including (1) 0.5 mM of ^15^N, ^13^C-labeled hHR23a; (2) 0.5 mM of ^15^N, ^13^C, 50% ^2^H-labeled hHR23a; and (3) 0.5 mM of ^13^C-labeled hHR23a. For the backbone assignments, 2D ^1^H-^15^N HSQC, 3D HNCA/HN(CO)CA, HNCO/HN(CA)CO, HNCACB/HN(CO)CACB spectra were recorded on sample 1 with a Varian INOVA 600 MHz spectrometer. For distance constraints, 2D ^1^H-^15^N HSQC and ^15^N-dispersed NOESY (200 ms mixing time) spectra were acquired with a Varian INOVA 800 MHz spectrometer on sample 2. 2D ^1^H-^13^C HSQC, 3D ^13^C-edited NOESY-HSQC (80 ms mixing time) and 2D NOESY (80 ms mixing time) spectra were acquired for the aliphatic and aromatic regions on sample 3. All experiments were conducted at 25°C in NMR buffer (20 mM NaPO_4_ at pH 6.5, 100 mM NaCl, 2 mM DTT, 0.1 % NaN_3_, and 10% ^2^H_2_O / 90% ^1^H_2_O), except for 2D NOESY and ^13^C-edited NOESY-HSQC experiments, which were acquired on sample 3 dissolved in 100% ^2^H_2_O.

All NMR data processing was performed with NMRpipe (Delaglio et al., 1995), and spectra were visualized and analyzed with XEASY (Bartels et al., 1995). Secondary structure was assessed by comparing chemical shift values of C_α_ and C’ atoms to random coil positions to generate a chemical shift index (CSI) (Wishart and Sykes, 1994).

### Extent of assignments and data deposition

**Figure 1:**
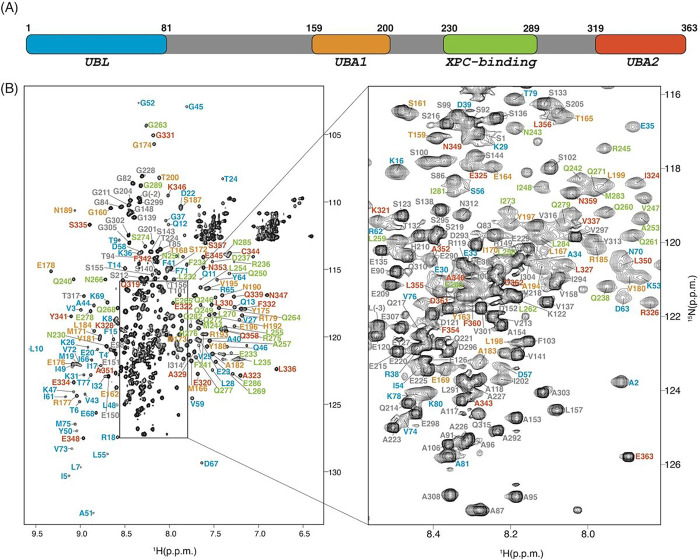
hHR23a domain layout and 2D ^1^H-^15^N HSQC spectrum **(A)** Domain organization of hHR23a with the UBL, UBA1, XPC-binding, and UBA2 domain shaded in blue, orange, green, and red, respectively. **(B)** 2D ^1^H-^15^N HSQC spectrum of ^15^N, ^13^C, 50% ^2^H-labeled hHR23a (0.5 mM) in 20 mM NaPO_4_ at pH 6.5, 100 mM NaCl, 2 mM DTT, 0.1% NaN_3_ and 10% ^2^H_2_O / 90% ^1^H_2_O collected at 25 °C on a Varian INOVA 800 MHz spectrometer. Residue type and sequence position are included for the signals corresponding to backbone amides and labeled in blue, orange, green, red, and grey for UBL, UBA1, XPC-binding, UBA2 domain, and linker region, respectively. A dashed box indicates the enlarged region shown in the panel on the right.

Figure 1 shows an assigned 2D ^1^H-^15^N HSQC spectrum of ^15^N, ^13^C, 50% ^2^H-hHR23a at pH 6.5 and 25°C. For amino acids within the four structural domains (M1-A81, T159-T200, N230-G289, and Q319-E363), 99.5% of backbone ^N^H atoms (missing M1), 94% of backbone N atoms (missing M1 and prolines), and 98% of backbone Cα, C’ and Hα atoms were assigned. 89% of sidechain aliphatic protons and 83% of sidechain aliphatic carbon atoms were assigned. The assignments have been deposited in the BioMagResBank (BMRB, http://bmrb.io) with accession code 52059.

**Figure 2:**
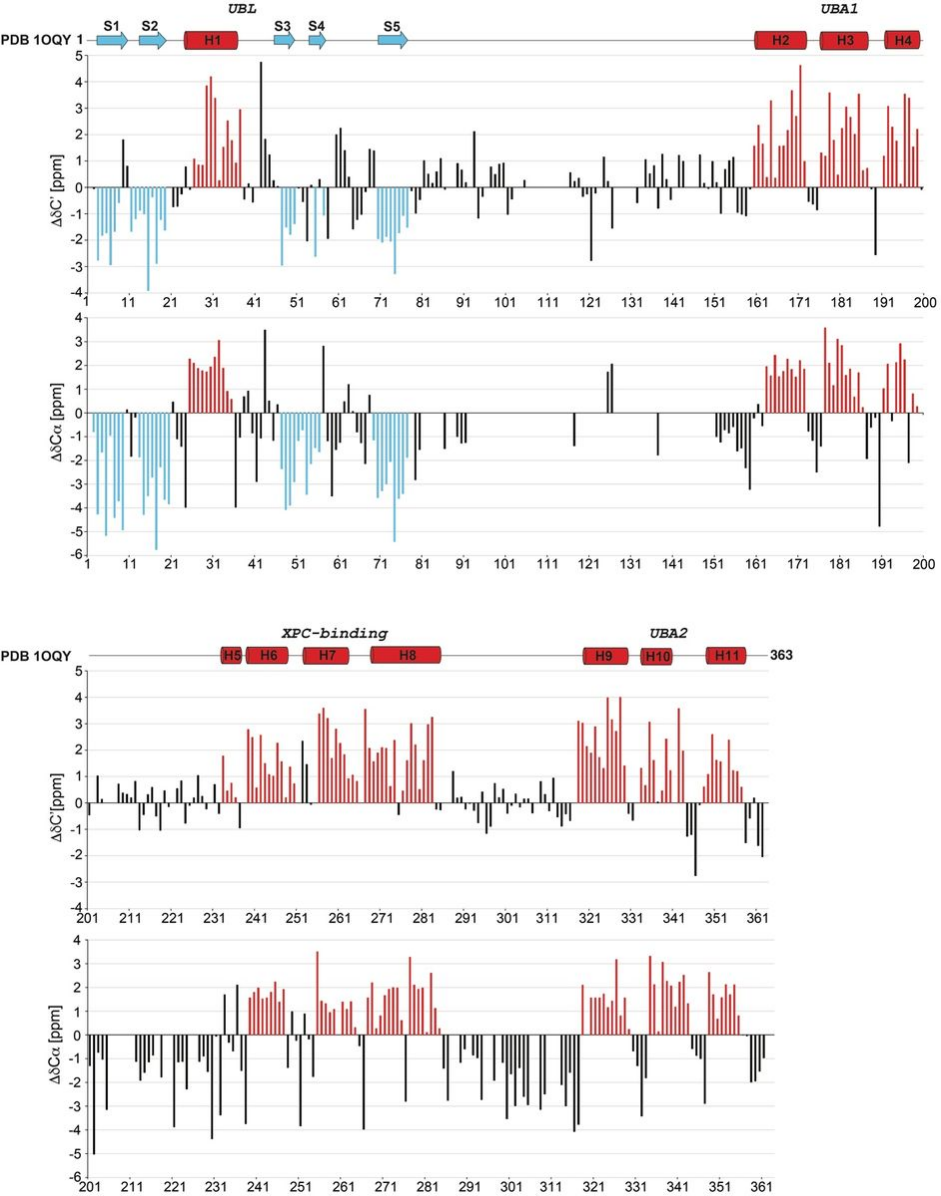
Secondary structure of the hHR23a obtained from the NMR structure 1OQY (top panel) or as predicted by DδC’ (middle panel) and DδCα (bottom panel) are displayed along the amino acid sequence. DδC’ and DδCα are calculated by subtracting randomly coiled values for the corresponding amino acid type (Wishart and Sykes, 1994) from hHR23a carbonyl and Cα atoms chemical shifts. The DδC’ and DδCα values predicted for β strands and α helices are colored in blue and red, respectively.

The secondary structure of the hHR23a was predicted by plotting the difference between the chemical shift values of carbonyl and Cα atoms relative to those of randomly coiled values (Wishart and Sykes, 1994) (Fig. 2). Five β strands and eleven α helices were predicted, consistent with the NMR structure of hHR23a (PDB 1OQY). All secondary structure was further confirmed by NOE interactions from ^15^N-edited NOESY-HSQC and ^13^C-edited NOESY-HSQC spectra.

## Data Availability

Assignments hHR23a have been deposited in the BMRB under accession code 52059. The plasmid for generation of hHR23a as used in this study is available upon request.

## References

[1] Bartels C., Xia T. H., Billeter M., Guntert P., & Wuthrich K. (1995). The program XEASY for computer-supported NMR spectral analysis of biological macromolecules. J. Biomol. NMR, 6(1), 1–10. doi:10.1007/BF00417486. 22911575

[2] Bertolaet B. L., Clarke D. J., Wolff M., Watson M. H., Henze M., Divita G., (2001). UBA domains of DNA damage-inducible proteins interact with ubiquitin. Nat. Struct. Biol., 8(5), 417–22. doi:10.1038/87575. 11323716

[3] Byeon I. L., Calero G., Wu Y., Byeon C. H., Jung J., DeLucia M., (2021). Structure of HIV-1 Vpr in complex with the human nucleotide excision repair protein hHR23A. Nat Commun, 12(1), 6864. doi:10.1038/s41467-021-27009-w. 34824204 PMC8617076

[4] Byeon I. L., Jung J., Byeon C. H., DeLucia M., Ahn J., & Gronenborn A. M. (2020). Complete (1)H, (13)C, (15)N resonance assignments and secondary structure of the Vpr binding region of hHR23A (residues 223–363). Biomol NMR Assign, 14(1), 13–17. doi:10.1007/s12104-019-09913-x. 31463759 PMC7047585

[5] Chen X., Htet Z. M., Lopez-Alfonzo E., Martin A., & Walters K. J. (2021). Proteasome interaction with ubiquitinated substrates: from mechanisms to therapies. FEBS J, 288(18), 5231–5251. doi:10.1111/febs.15638. 33211406 PMC8131406

[6] Chen X., Randles L., Shi K., Tarasov S. G., Aihara H., & Walters K. J. (2016). Structures of Rpn1 T1:Rad23 and hRpn13:hPLIC2 Reveal Distinct Binding Mechanisms between Substrate Receptors and Shuttle Factors of the Proteasome. Structure, 24(8), 1257–70. doi:10.1016/j.str.2016.05.018. 27396824 PMC4972676

[7] Chen X., & Walters K. J. (2012). Identifying and studying ubiquitin receptors by NMR. Methods Mol. Biol., 832, 279–303. doi:10.1007/978-1-61779-474-2_20. 22350893 PMC6392013

[8] Dantuma N. P., Heinen C., & Hoogstraten D. (2009). The ubiquitin receptor Rad23: at the crossroads of nucleotide excision repair and proteasomal degradation. DNA Repair (Amst), 8(4), 449–60. doi:10.1016/j.dnarep.2009.01.005. 19223247

[9] Delaglio F., Grzesiek S., Vuister G. W., Zhu G., Pfeifer J., & Bax A. (1995). NMRPipe: a multidimensional spectral processing system based on UNIX pipes. J. Biomol. NMR, 6(3), 277–93. http://www.ncbi.nlm.nih.gov/pubmed/8520220. 8520220 10.1007/BF00197809

[10] Dieckmann T., Withers-Ward E. S., Jarosinski M. A., Liu C. F., Chen I. S., & Feigon J. (1998). Structure of a human DNA repair protein UBA domain that interacts with HIV-1 Vpr. Nat Struct Biol, 5(12), 1042–7. doi:10.1038/4220. 9846873

[11] Elsasser S., Gali R. R., Schwickart M., Larsen C. N., Leggett D. S., M\ ller B., (2002). Proteasome subunit Rpn1 binds ubiquitin-like protein domains. Nat. Cell Biol., 4(9), 725–30. doi:10.1038/ncb845. 12198498

[12] Gomez T. A., Kolawa N., Gee M., Sweredoski M. J., & Deshaies R. J. (2011). Identification of a functional docking site in the Rpn1 LRR domain for the UBA-UBL domain protein Ddi1. BMC Biol., 9(1), 33. doi:10.1186/1741-7007-9-33. 21627799 PMC3126750

[13] Husnjak K., Elsasser S., Zhang N., Chen X., Randles L., Shi Y., (2008). Proteasome subunit Rpn13 is a novel ubiquitin receptor. Nature, 453(7194), 481–8. doi:10.1038/nature06926. 18497817 PMC2839886

[14] Jiang W. X., Gu X. H., Dong X., & Tang C. (2017). Lanthanoid tagging via an unnatural amino acid for protein structure characterization. J Biomol NMR, 67(4), 273–282. doi:10.1007/s10858-017-0106-9. 28365903

[15] Kamionka M., & Feigon J. (2004). Structure of the XPC binding domain of hHR23A reveals hydrophobic patches for protein interaction. Protein Sci, 13(9), 2370–7. doi:10.1110/ps.04824304. 15322280 PMC2280024

[16] Kang Y., Chen X., Lary J. W., Cole J. L., & Walters K. J. (2007a). Defining how ubiquitin receptors hHR23a and S5a bind polyubiquitin. J Mol Biol, 369(1), 168–76. doi:10.1016/j.jmb.2007.03.008. 17408689 PMC3864866

[17] Kang Y., Zhang N., Koepp D. M., & Walters K. J. (2007b). Ubiquitin receptor proteins hHR23a and hPLIC2 interact. J Mol Biol, 365(4), 1093–101. doi:10.1016/j.jmb.2006.10.056. 17098253 PMC1994665

[18] Mueller T. D., & Feigon J. (2002). Solution structures of UBA domains reveal a conserved hydrophobic surface for protein-protein interactions. J Mol Biol, 319(5), 1243–55. doi:10.1016/S0022-2836(02)00302-9. 12079361

[19] Mueller T. D., & Feigon J. (2003). Structural determinants for the binding of ubiquitin-like domains to the proteasome. EMBO J., 22(18), 4634–45. doi:10.1093/emboj/cdg467. 12970176 PMC212733

[20] Osei-Amponsa V., & Walters K. J. (2022). Proteasome substrate receptors and their therapeutic potential. Trends Biochem Sci, 47(11), 950–964. doi:10.1016/j.tibs.2022.06.006. 35817651 PMC9588529

[21] Rosenzweig R., Bronner V., Zhang D., Fushman D., & Glickman M. H. (2012). Rpn1 and Rpn2 coordinate ubiquitin processing factors at proteasome. J. Biol. Chem., 287(18), 14659–71. doi:10.1074/jbc.M111.316323. 22318722 PMC3340268

[22] Shi Y., Chen X., Elsasser S., Stocks B. B., Tian G., Lee B. H., (2016). Rpn1 provides adjacent receptor sites for substrate binding and deubiquitination by the proteasome. Science, 351(6275). doi:10.1126/science.aad9421. PMC4980823 26912900

[23] Walters K. J., Lech P. J., Goh A. M., Wang Q., & Howley P. M. (2003). DNA-repair protein hHR23a alters its protein structure upon binding proteasomal subunit S5a. Proc. Natl. Acad. Sci. U. S. A., 100(22), 12694–9. doi:10.1073/pnas.1634989100. 14557549 PMC240680

[24] Wang Q., Goh A. M., Howley P. M., & Walters K. J. (2003). Ubiquitin recognition by the DNA repair protein hHR23a. Biochemistry, 42(46), 13529–35. doi:10.1021/bi035391j. 14621999

[25] Wishart D. S., & Sykes B. D. (1994). The 13C chemical-shift index: a simple method for the identification of protein secondary structure using 13C chemical-shift data. J Biomol NMR, 4(2), 171–80. https://www.ncbi.nlm.nih.gov/pubmed/8019132. 8019132 10.1007/BF00175245

[26] Withers-Ward E. S., Mueller T. D., Chen I. S., & Feigon J. (2000). Biochemical and structural analysis of the interaction between the UBA(2) domain of the DNA repair protein HHR23A and HIV-1 Vpr. Biochemistry, 39(46), 14103–12. doi:10.1021/bi0017071. 11087358

[27] Yasuda S., Tsuchiya H., Kaiho A., Guo Q., Ikeuchi K., Endo A., (2020). Stress- and ubiquitylation-dependent phase separation of the proteasome. Nature, 578(7794), 296–300. doi:10.1038/s41586-020-1982-9. 32025036

